# Deep HT: A deep neural network for diagnose on MR images of tumors of the hand

**DOI:** 10.1371/journal.pone.0237606

**Published:** 2020-08-14

**Authors:** Xianliang Hu, Zongyu Liu, Haiying Zhou, Jianyong Fang, Hui Lu

**Affiliations:** 1 School of Mathematical Sciences, Zhejiang Univeristy, Hangzhou, Zhejiang Province, P. R. China; 2 Department of Orthopedics, The First Affiliated Hospital, Zhejiang University, Hangzhou, Zhejiang Province, P. R. China; 3 Suzhou Warrior Pioneer Software Co., Ltd., Suzhou, Jiangsu Province, P. R. China; Newcastle University, UNITED KINGDOM

## Abstract

**Background:**

There are many types of hand tumors, and it is often difficult for imaging diagnosticians to make a correct diagnosis, which can easily lead to misdiagnosis and delay in treatment. Thus in this paper, we propose a deep neural network for diagnose on MR Images of tumors of the hand in order to better define preoperative diagnosis and standardize surgical treatment.

**Methods:**

We collected MRI figures of 221 patients with hand tumors from one medical center from 2016 to 2019, invited medical experts to annotate the images to form the annotation data set. Then the original image is preprocessed to get the image data set. The data set is randomly divided into ten parts, nine for training and one for test. Next, the data set is input into the neural network system for testing. Finally, average the results of ten experiments as an estimate of the accuracy of the algorithm.

**Results:**

This research uses 221 images as dataset and the system shows an average confidence level of 71.6% in segmentation of hand tumors. The segmented tumor regions are validated through ground truth analysis and manual analysis by a radiologist.

**Conclusions:**

With the recent advances in convolutional neural networks, vast improvements have been made for image segmentation, mainly based on the skip-connection-linked encoder decoder deep architectures. Therefore, in this paper, we propose an automatic segmentation method based on DeepLab v3+ and achieved a good diagnostic accuracy rate.

## 1. Introduction

In medical treatment, a radiograph of the hand is mandatory for all lesions. Tumors of the hand comprise a vast array of lesions involving skin, soft tissue and bone. Several major types of hand tumor are benign, and malignant tumors are also appeared in specific area with its specific character. 69.2% -95% of tumors of the hand do not involve cutaneous malignancy are benign [1, 2]. Some benign growths may not need excision. Ganglions, giant cell tumors, granulomas, Lipoma, and hemangiomas are the top five tumors with the highest incidence rate. The patient’s history, physical examination, radiography and laboratory examinations are essential factors for diagnosis of hand tumors. Radiograph is capable of detecting and characterizing hand tumors. Size, localization and characteristic signal are significant in radiography diagnosis.

**Fig 1:**
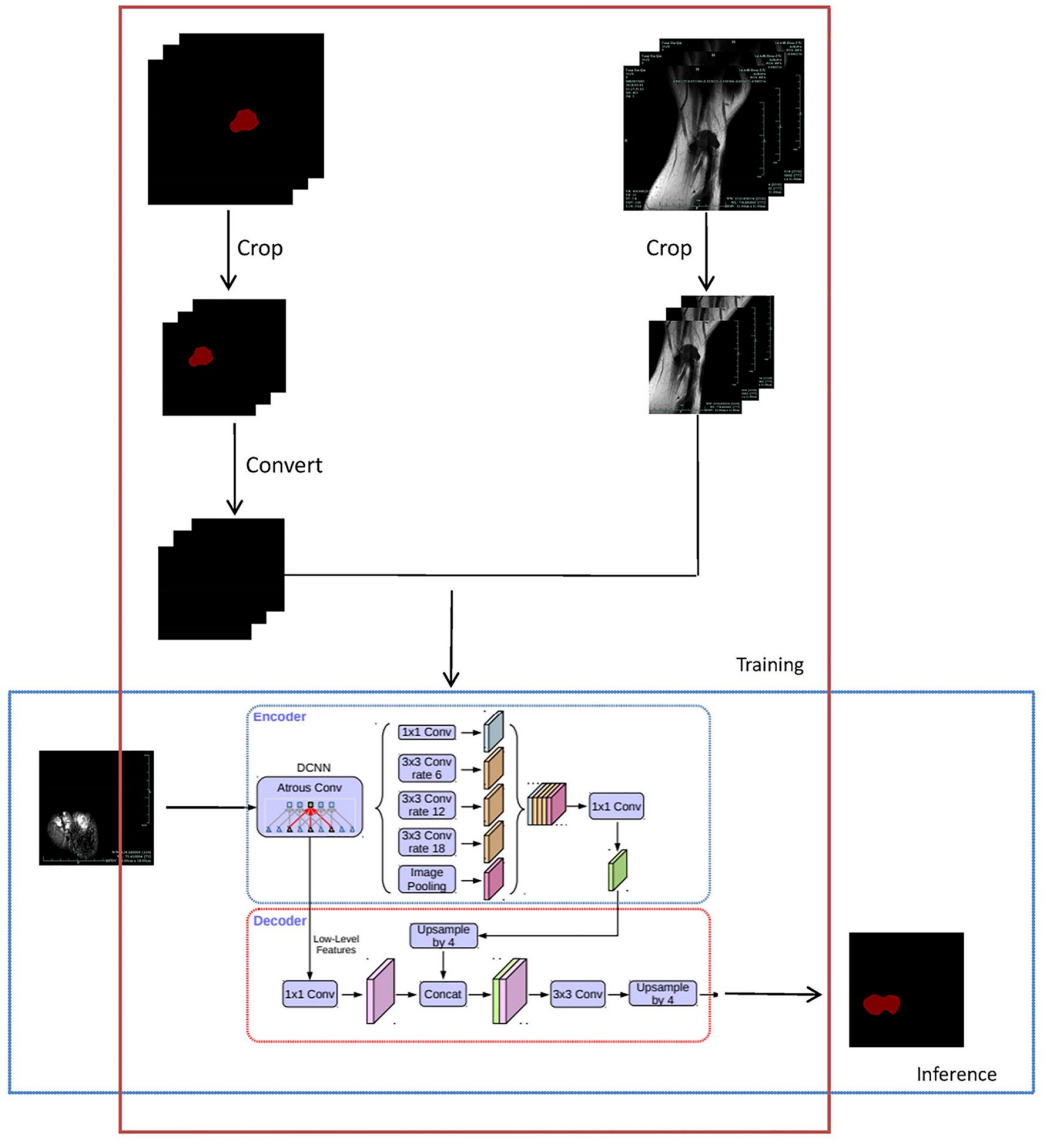
Operation flow chart of DeepHandTumor. In the training stage, firstly, we divide the data set into a training set and a validation set. Then crop the input image to a uniform size and convert the annotation image into a grayscale image, input them into the model together; For inference stage, input the picture into the model and get the diagnosis result.

Magnetic resonance imaging (MRI) would be an excellent choice, as it clearly demonstrates the anatomical structure and tumor characteristics [3–5] (Fig 1). It can allow doctors to generate a clinically differential diagnosis based on the distinguishing features of hand tumors [6, 7]. Failure to diagnose glomus tumors of the hand on MRI is associated with smaller tumor size, atypical glomus tumor pathology and atypical location [8]. Small tumor size and atypical location cause difficulties in clinical diagnosis of hand tumors [2, 9]. The differential diagnosis of hand tumors is challenging and important for clinician to select the proper treatment [10–12]. Recurrence of tumor in the hand affects nerve and tendon function, leading to hand deformities. This not only causes inconvenience to the patient's life and work, but also affects his image and social activities [13, 14]. The preoperative diagnosis is very important. Artificial intelligence can provide a reference for clinicians. This will reduce the country's medical expenses, reduce the financial burden of patients, and most importantly, give patients a good prognostic function [15, 16].

Medical image analysis develops various methods to solve medical images and their application in clinical care. Among these methods and applications, automatic image segmentation plays an important role in treatment planning, disease diagnose, and pathology learning strategies [17–20].

In recent years, deep learning-based algorithms, especially Convolutional Neural Network(CNN), have promoted significant advances in medical image analysis [21]. The foremost appeal of CNN is its ability to learn increasingly complicated features from the input data. For instances, architectures of CNN such as, Alex Krizhevsky network(AlexNet), launched the current deep learning boom by winning the 2012 ILSVRC competition by a huge margin [22, 23]. With the successful use of CNNs for image recognition, Simonyan et al. proposed a simple and effective design principle for CNN architectures. Their architecture named as VGG, it popularizes the idea of using smaller filter kernels and deeper networks (up to 19 layers for VGG19, compared to 7 for AlexNet) [24]. At the same time, GoogLeNet won the 2014 ILSVRC competition and is also known as Inception-V1 [25]. GoogLeNet also popularizes the idea of not using fully-connected layers at the end, but rather global average pooling, significantly reducing the number of model parameters. To address the problem faced during training of deeper nets. In 2015, He et al. proposed ResNet in which they exploited the idea of bypass pathways used in highway networks [26], (having 20 and 8 times more depth than AlexNet and VGG, respectively) won the 2015-ILSVRC championship. Even with increased depth, ResNet exhibited lower computational complexity than VGG [24]. Deep learning methods are increasingly used to improve clinical practice, and the list of examples is long, growing daily. In image segmentation, a common feature in almost all state-of-the-art methods is the encoder-decoder architecture with skip connections [17]. The encoder module captures high-level semantic information using down sampling and convolution operations; the decoder module gradually recovers spatial information using techniques such as deconvolution or upsampling, while skip connections are utilized to pass the low-level texture and location information.

In this research, CNN based system for tumor segmentation is proposed. Since, most of the DeepLab v3+ based segmentation focus on non-medical applications, this paper provides an insight into the possibility of using DeepLab v3+ in medical imaging as well [27, 28]. The segmented tumor regions are validated through ground truth analysis and analyzation process was done by different radiologist.

Finally, since not all pixels are of equivalent difficulty in segmentation, we introduce a weighted cross-entropy loss to assist the network to pay more attention to the segmentation targets.

## 2. Materials

### 2.1 Description of the dataset

**Fig 2:**
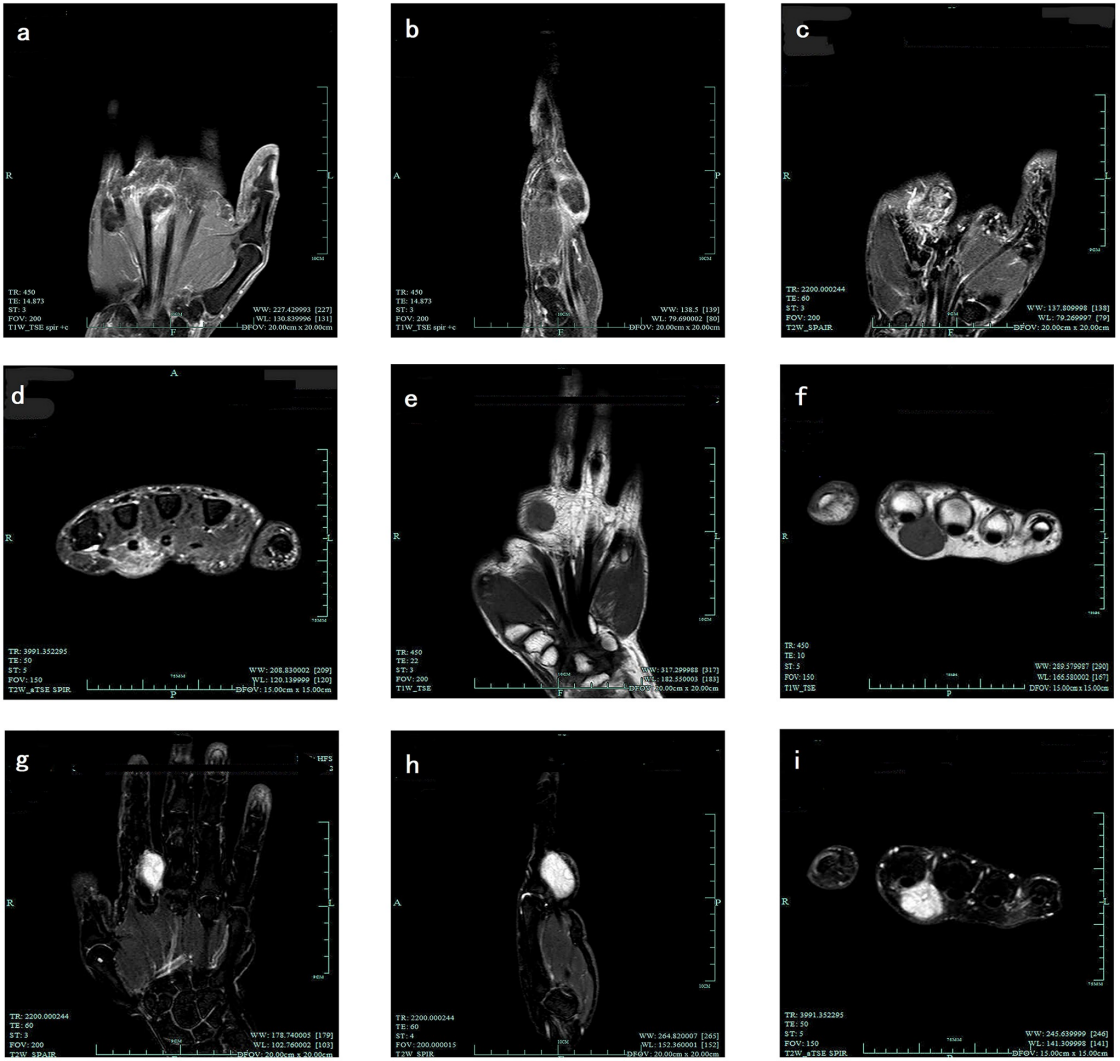
(a) soft tissue chondroma in T1WI coronal section; (b) soft tissue chondroma in T1WI sagittal section; (c) soft tissue chondroma in T2WI coronal section; (d) soft tissue chondroma in T2WI transverse section; (e) angioleiomyoma in T1WI coronal section; (f) angioleiomyoma in T1WI transverse section; (g) angioleiomyoma in T2WI coronal section; (h) angioleiomyoma in T2WI sagittal section; (i) angioleiomyoma in T2WI transverse section.

In this study, we collected MRI from 221 different patients of hand tumor. In this research, MRI hand tumor dataset was used. These MRI images were collected from The First Affiliated Hospital, Zhejiang University, from 2016 to 2019. This particular dataset consisted of labels, patient IDs, image data and tumor boarder coordinates along with T1- weighted axial, sagittal and coronal planes. It consisted of 221 MRIs from 221 patients with five kinds of hand tumors; namely Deep HT (deep hand tumor), including include the ganglion (87 cases), giant cell tumor of tendon sheath(54 cases), lipoma(44 cases), hemangioma(26 cases) and schwannoma(10 cases), which were also the top five tumor with the highest incidence rate (Fig 2(a)–2(i)). Considering the size of datasets was small and the inability to conduct clinical trials, in order to obtain reliable experimental results, we used cross-validation methods for experiments.

### 2.2 Data preparation

We had noticed that Deeplab v3+ had reached state of the art in VOC2012, Cityscapes, ADE20K and other public datasets, but the segmentation targets in these datasets were mostly pedestrians, streets, houses, which accounted for a large proportion in the image, and the frequency of segmentation targets was not so much different. The dataset of medical images was quite different from the standard datasets. Direct use of existing models did not yield satisfactory results.

The machine we used was Philips Achieva 1.5T (Netherlands). All kinds of MRI imagines are chosen for this study, including sagittal and coronal planes, T1 axial, T2 axial and contrast-enhanced MRI. Medical images like MRI almost have a feature of low-contrast and high-noise. We used contrast limited adaptive histogram equalization (CLAHE) to process the original MRI first. CLAHE was wildly applied in the variant medical image given that it could improve contrast while limited the amplification of noise.

We naturally resized all images to a fixed size 513 × 513 (padding small images, cropping large images randomly), uniformly sized images could be used for fast batch training. Resizing would not affect the training results, because image segmentation was a task of classifying pixel, and had nothing to do with the size and ratio of the image.

**Fig 3:**
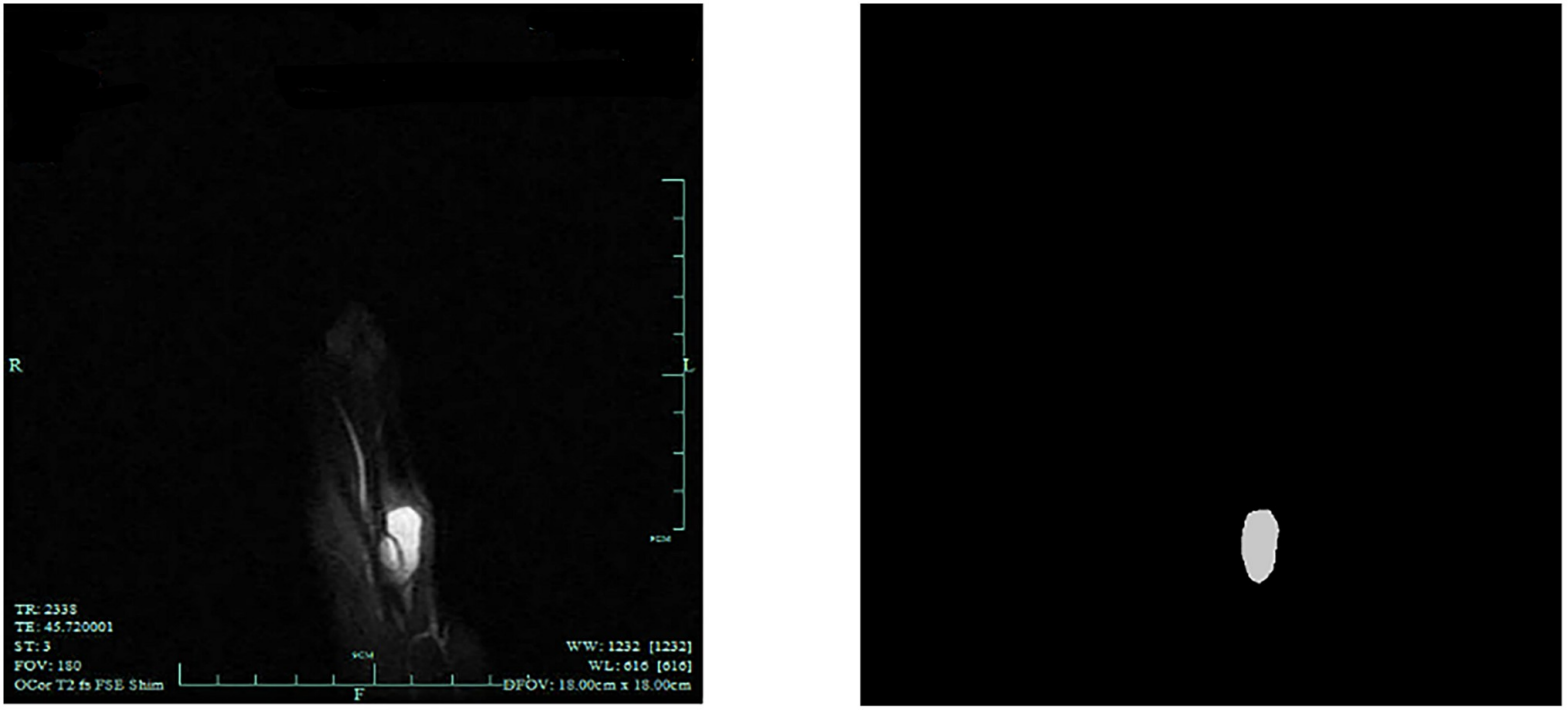
The enhanced image with tumor and the label mask.

Finally, we transcoded the 16-bit color annotation masks to 8-bit grayscale annotation masks, used for model training (Fig 3).

### 2.3 Data augmentation

We chose data augmentation according to the characteristics of our data. When shooting hand tumor MRI, patients’ hands would not be scanned upside down, their fingertips were always facing up, thus the flip of the image should be horizontal. In the meantime, patients might swing a little bit when scanned, so a random small angle rotation was needed. Different types of tumors had different shapes and did not have an affine transformation relationship, so we also elastically deformed the data. Considering that brightness and contrast were optimized in CLAHE, our augmentation mainly included random deformation like slight rotation, horizontal flip and elastically deform. Under the premise of retaining the complete hand, the original image was equally scaled, after the above steps we obtained 900 training set and 80 validation set.

### 2.4 Dividing the dataset

Firstly, we randomly divided the dataset into ten parts, using nine of them in turn as the training set and one as the test set. Next, we augmented the training set and conducted experiments. Finally, we averaged the results of ten experiments as an estimate of the accuracy of the algorithm.

## 3 Methods

### 3.1 Patient and public involvement

In this study, the MRI images of patients with different hand tumor were collected from The First Affiliated Hospital, Zhejiang University, from 2016 to 2019, all patients were accepted follow-up in our outpatient clinic. The choice of the required image should meet the requirement that the tumor image is a hand tumor, single or multiple, and the final pathological result is a benign tumor. While the MRI image of patients with previous hand trauma history, hand deformity, hand infection, hand soft tissue or bone defect, or multiple tumors in which the pathological type of the tumor is confirmed as different types of tumors, or the pathological type of the hand tumor is malignant tumor, will be excluded. Then, after preprocessing, the image data set was randomly divided into training set and test set. Ethical approval was given by the medical ethics committee of the First Affiliated Hospital, College of Medicine, Zhejiang University, written informed consent was obtained for each patient, and authors had access to information that could identify individual participants during or after data collection.

### 3.2 Framework of the data flow

As we mentioned earlier, hand tumor segmentation requires a clearer boundary than traditional segmentation tasks.

**Fig 4:**
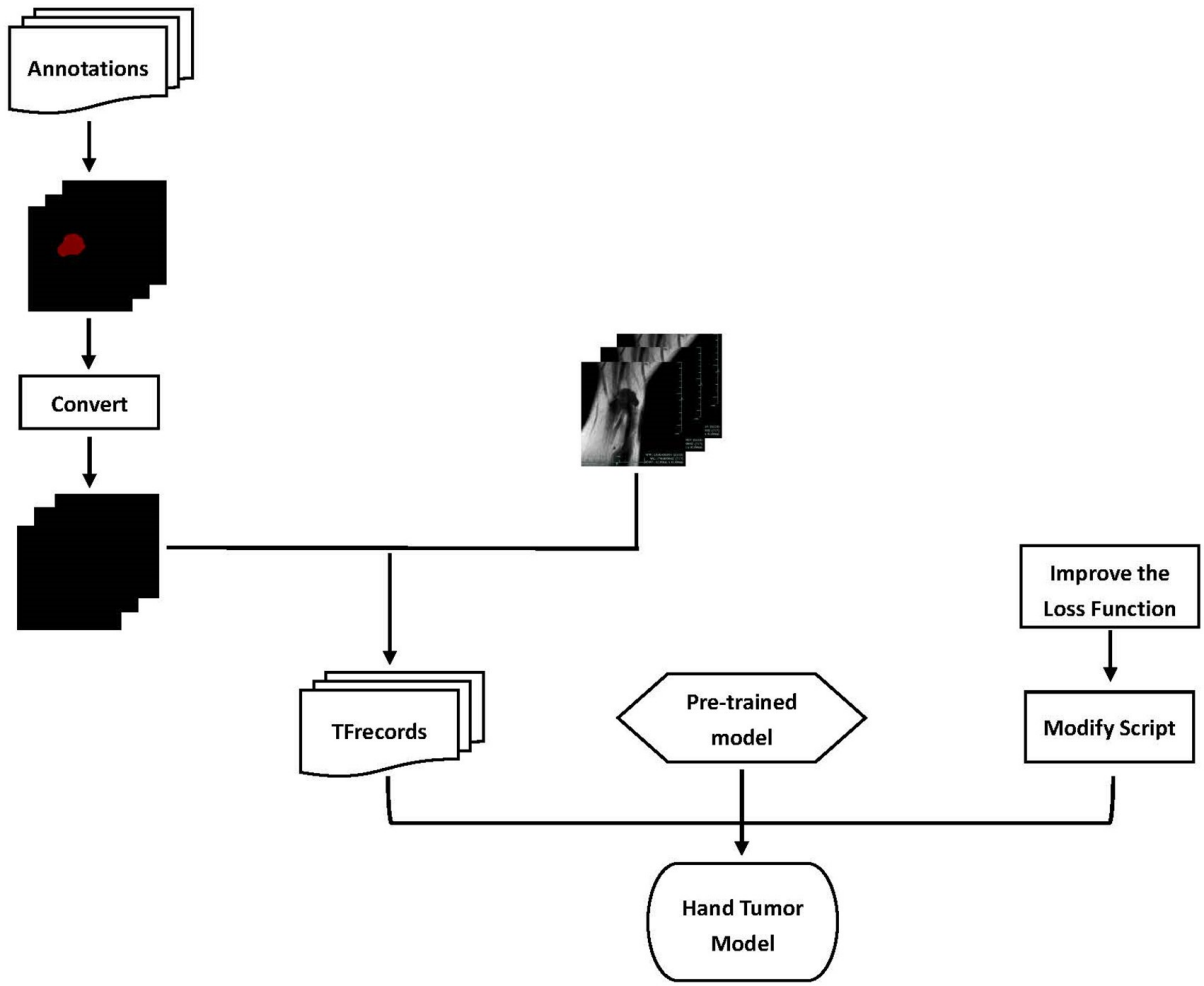
A system overview for the data flow. TFrecords format dataset is more convenient for model reading, using improved loss functions and pre-trained models, we can train more accurate Hand Tumor Model.

A systematic overview for the proposed methods is plotted in the Fig 4. Firstly, we invited two experienced radiologists who didn’t know the results of the MRI images to label the tumor images we provided using the software “labelme”, got the annotation files, and unified the annotation results from different radiologists to form the final labeled datasets. Secondly, we used the pre-processing script to preprocess the annotation files, and generated grayscale annotation masks. Then, we packaged the training sets and tested sets to generate TFRecord files. Thirdly, we inputted the TFRecord files into the pre-training model, and the loss function was used to iteratively train to obtain the automatic segmentation model. After the model training was completed, the tumor image was predicted by the prediction script, or the tumor image was subjected to batch segmentation prediction using the verification script.

### 3.3 Deep neural network model

**Fig 5:**
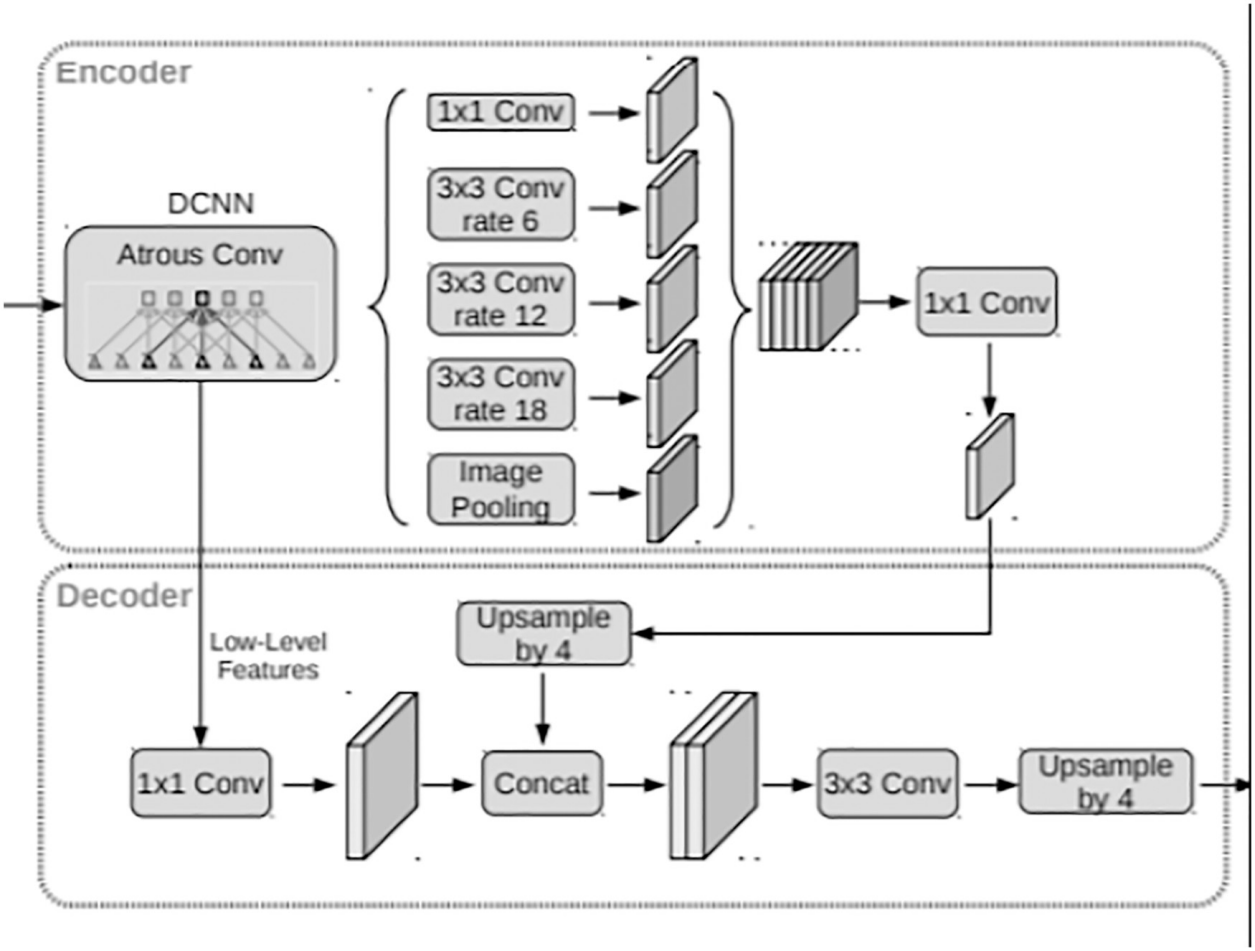
Network structure of Deeplab v3+.

DeepLab v3+ propose a novel encoder-decoder structure [28] (Fig 5). The Encoder module gradually reduces the resolution of the feature map and captures high-level semantic information; the Decoder module gradually recovers spatial information using techniques such as deconvolution or upsampling.

First, we inputted image into the improved Xception network extraction feature to get the low-level coding feature map [29].

In the encoder module, DeepLabv3+ used multiple parallel expansion convolutions (i.e., Atrous Spatial Pyramid Pooling(ASPP)) to generate feature maps with multi-scale information, and concatenated the feature maps to obtain the high-level coding feature map [30].

In the decoder module, firstly, we performed 4-fold bilinear upsampling on the high-level coding feature map, secondly we concatenated the upsampled feature map with the low-level coding feature map from the encoder module, thirdly we passed the concatenated feature map to a 3×3 convolution to refine feature, finally we used 4 times bilinear upsampling on the refined feature map to obtain a predicted image. The predicted result of the network output was the softmax value at the pixel level, that was, the value of each pixel was

$$(p_{1}(x),p_{2}(x),...,p_{K}(x)),p_{k}(x)=\frac{\text{exp}(a_{k}(x))}{\sum_{i=1}^{K}\text{exp}(a_{i}(x))},$$

Indicating the probability that the pixel point x in the predicted image belonged to the target category i.

The target of finding a tumor was in principle binary, saying each pixel was either tumor or not. The cross-entropy loss function for all pixels in the perspective of classification was reasonable. Our label for tumor showed that the area of tumor and background were out of proportion. Only a small part of pixels was hand tumor where others are all background. Thus original cross entropy would guide the model tend to predict every pixel as background while getting little more loss. We used weighted cross entropy loss, where wl(x) corresponded to the weight of different labels (background or tumor), l(x) was the label type of pixel point x.

The expression of the loss function was as follows

$$L(x)=\sum_{j=1}^{batch\_size}\sum_{x}w_{l}{}_{{}_{\left(x\right)}}(x)\text{log}(p_{l_{\left(x\right)}}(x))$$

## 4 Experimental results

The segmentation quality is measured by the mean intersection-over-union (mIoU) score, the pixel accuracy and the mean accuracy over all classes.

Firstly, we directly used the existing Deeplab v3+ model to conduct experiments on the tumor data set, which achieved only 37.7% accuracy. This experiment proves that our previous inferences are correct, the dataset of medical images was quite different from the standard datasets. Direct use of existing models did not yield satisfactory results.

**Fig 6:**
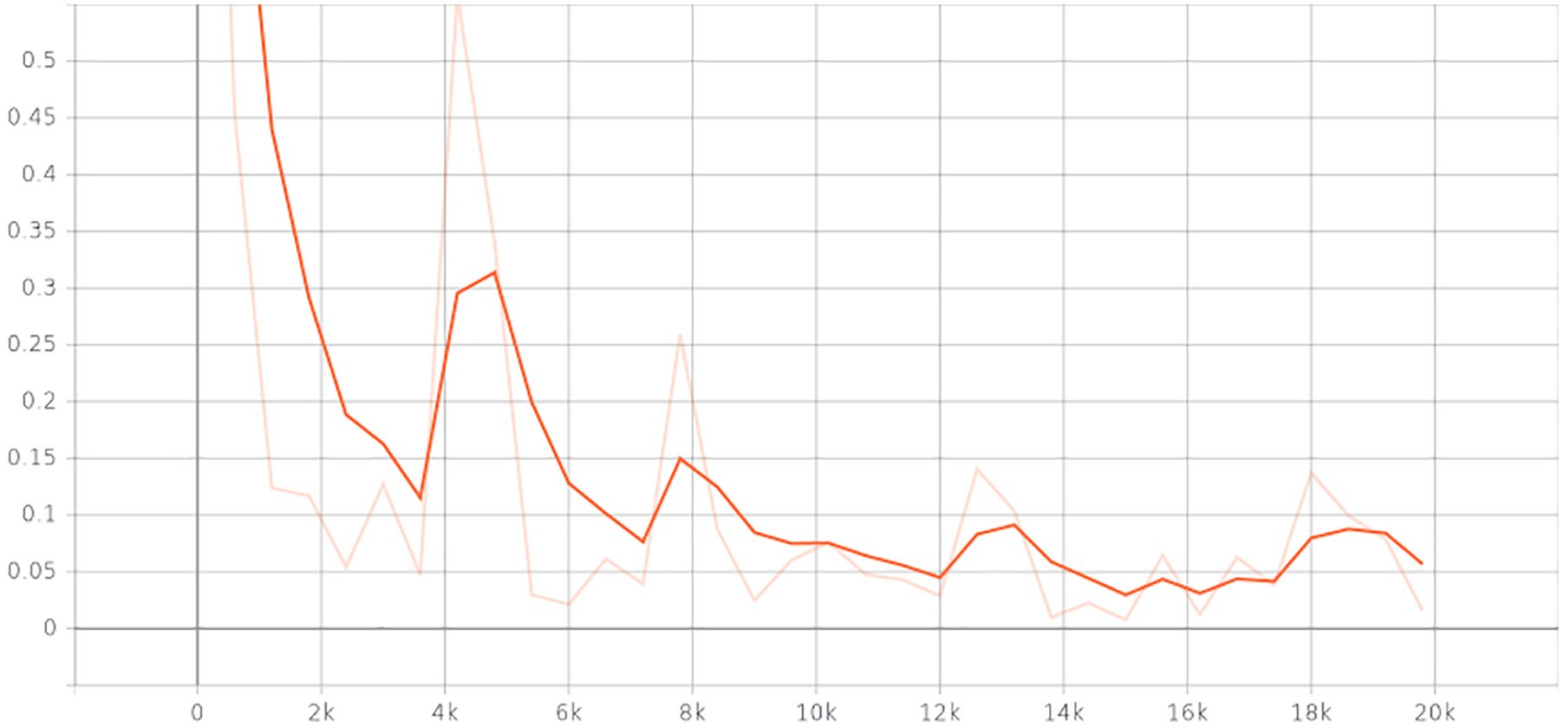
Change curve of loss function in a cross-validation experiment.

To show the effectiveness of our approach, we conducted a five-fold crossover experiment on the data set. Every experiment we get 496 training set and 123 validation set. The results of the five cross-validation experiments are shown in the Table 1. The figure shows the change curve of loss function in an experiment (Fig 6).

**Table 1:** The results of fivecrossvalidation experiments, due to the randomness of the optimization process, there is an error of about 0.1 in the experimental results.

Cross-validation experiment	mIoU
First Fold	0.698
Second Fold	0.708
Third Fold	0.691
Forth Fold	0.687
Fifth Fold	0.716

In this work, we implemented our model’s training, evaluation, error analysis, and visualization pipeline using TensorFlow, which is a popular deep learning framework, and then compiled using cuDNN computational kernels. We used stochastic gradient descent with momentum to optimize the loss of function. We set the initial learning rate to 0.0001, learning rate decay factor to 0.0005, and decay step size to 2,000. Instead of using a fixed number of steps, we trained our model until the mean average precision of the training set converged, and then evaluated the model using the validation set. We used three NVIDIA Tesla P4 GPU for all experiments.

## 5 Discussion & conclusions

The patient’s history, physical examination, radiography and laboratory examinations are essential factors for diagnosis of hand tumors. The hand is a complex anatomic region, including bone, joints and soft tissues with muscles, vessels, and nerves. Plain x-ray is sensitive to distinguish bony tissue, CT scan is sensitive to finer bony tissue, ultrasound and MRI is sensitive to soft-tissue. Magnetic resonance imaging without bone artifacts, can be directly used for multi-directional (transverse, coronal, sagittal or any angle) section, showing the anatomy and lesions of soft tissue tumors. Its "flowing effect" can display vascular structures without angiographic contrast agents, so it shows uniqueness in the "no damage" of blood vessels, and the mutual identification of tumors, tendons and their vascular structures [31–33].

Image segmentation has an almost 50 years long history, several traditional machine learning methods have been made for MRI segmentation and tissue classification problems [34–37].

MRI segmentation using deep learning approaches, typically CNNs, is now penetrating the whole field of applications. For instances, Fully Convolutional Networks(FCN) is a milestone in semantic segmentation [38]. It first proposes the idea of pixel-level classification of images, and converts the fully connected layers in traditional CNN into convolutional layers, which can accept input images of any size. U-Net continues the idea of full convolutional layer, and introduces the encoder-decoder structure [39]. In addition, based on the ideas of ResNet, there are skip connections between encoder module and decoder module. A similar approach is used by V-Net proposed an extension to the U-Net, it is a three-dimensional version of U-net with volumetric convolutions and skip connections as in ResNet [40].

The prospective of Deep learning for tumor segmentation is at preliminary investigation stage and poorly studied. The primary challenges for medical image segmentation mainly lie in three aspects, (1) Complex boundary interactions, (2) Large appearance variation, (3) Low tissue contrast (Encoder-Decoder segmentation).

But we believe that the automatic segmentation technology has a bright future. Currently, this technology can be applied in the following aspects.

1. Assisting inexperienced doctor diagnosis
  Medical image segmentation is an important step in medical diagnosis and treatment. Only when the lesion is completely marked, the doctor can make a correct medical diagnosis based on the characteristics of the lesion and the patient information. In the real world, manual labeling not only takes a lot of time and effort, but also requires professional level medical knowledge as the basis, which poses a challenge to doctors with less experience. When the automatic segmentation technology is mature, we can package the algorithm into computer software. By connecting with the hospital case database, the lesions are automatically segmented during diagnosis and treatment, providing a reference for doctors to better reduce the rate of misdiagnosis.
  At this stage, the algorithm has the recognition level of professional doctors, our model accuracy rate is up to 71.6% (mIOU) In the future, as the data set increases, the accuracy of the model will be further improved.
2. Patient self-medication
  At this stage, the distribution of medical resources in various localities is uneven, and high-quality medical resources are concentrated in first-tier cities such as Beijing and Shanghai. At the same time, rural areas are relatively backward compared to urban medical conditions. When encountering major diseases such as cancer, people cannot seek medical treatment nearby. The treatment of cancer is an urgent task, and delaying the precious treatment time may lead to the spread of tumors and malignant lesions. Therefore, leting people self-diagnosis in the presence of discomfort, timely judgment of the physical condition, plays a vital role in the treatment of tumor diseases. Our algorithm can be packaged into a mobile app for people to download and install. When the user is unwell, he can use the mobile phone application to scan the MRI image to make a preliminary judgment on his own physical condition, so as to achieve early treatment and early recovery.

This is conducive to the treatment of patients, reducing the cost of medical expenses in the country and reducing the economic burden on patients.

However, there are still some shortcomings in our study, such as the small variety of hand tumors included, the insufficient sample size, and the relatively homogeneous number of participating medical centers. This is inevitable at the initial stage of the research, but in the future, we hope that based on the existing data and research results, we can realize the development of a multicenter, multi-category image recognition and diagnosis system for hand tumors, and we hope to inspire other researchers about similar healthy topic to promote the contribution of medicine to society and human health.

## Supporting information

S1 Algorithm The code and related information for the algorithm used is already open source at this website: https://github.com/AllenLau9679/DeepTumor. (TXT)

## Abbreviations

Deep HT: Deep hand tumor
MRI: Magnetic resonance imaging
